# VEGFR2 targeted microbubble-based ultrasound molecular imaging improving the diagnostic sensitivity of microinvasive cervical cancer

**DOI:** 10.1186/s12951-023-01984-2

**Published:** 2023-07-12

**Authors:** Junlin Zhong, Manting Su, Ye Jiang, Licong Huang, Ying Chen, Zhuoshan Huang, Xinling Zhang

**Affiliations:** 1 Department of Ultrasound, The Third Affiliated Hospital of Sun Yat-sen University, No. 600 Tianhe Road, Guangzhou, 510630 Guangdong China; 2 Department of Pathology, The Third Affiliated Hospital of Sun Yat-sen University, No. 600 Tianhe Road, Guangzhou, 510630 Guangdong China; 3 Department of Cardiovascular Medicine, The Third Affiliated Hospital of Sun Yat-sen University, No. 600 Tianhe Road, Guangzhou, 510630 Guangdong China

**Keywords:** Microinvasive cervical cancer, Noninvasive diagnosis, Molecular ultrasound imaging, VEGFR2

## Abstract

**Background:**

The current diagnostic methods of microinvasive cervical cancer lesions are imaging diagnosis and pathological evaluation. Pathological evaluation is invasive and imaging approaches are of extremely low diagnostic performance. There is a paucity of effective and noninvasive imaging approaches for these extremely early cervical cancer during clinical practice. In recent years, ultrasound molecular imaging (USMI) with vascular endothelial growth factor receptor type 2 (VEGFR2) targeted microbubble (MB_VEGFR2_) has been reported to improve the early diagnosis rates of breast cancer (including ductal carcinoma in situ), pancreatic cancer and hepatic micrometastases. Herein, we aimed to assess the feasibility of MB_VEGFR2_-based USMI in extremely early cervical cancer detection to provide an accurate imaging modality for microinvasive cervical cancer (International Federation of Gynecology and Obstetrics (FIGO) Stage IA1 and IA2).

**Results:**

We found MB_VEGFR2_-based USMI could successfully distinguish extremely early lesions in diameter < 3 mm from surrounding normal tissues (all *P* < 0.05), and the sensitivity gradually decreased along with increasing tumor diameter. Moreover, normalized intensity difference (NID) values showed a good linear correlation with microvessel density (MVD) (R^2^ = 0.75). In addition, all tumors could not be identified from surrounding muscles in subtracted ultrasound images when mice were administered MB_Con_.

**Conclusions:**

Overall, MB_VEGFR2_-based USMI has huge potential for clinical application for the early detection of microinvasive cervical cancer (FIGO Stage IA1 and IA2), providing the foothold for future studies on the imaging screening of this patient population.

**Supplementary Information:**

The online version contains supplementary material available at 10.1186/s12951-023-01984-2.

## Introduction

Globally, cervical cancer is the second most frequent gynecologic cancer and constitutes one of the most common cancers death among females, especially in underdeveloped countries [1]. According to the 2018 International Federation of Gynecology and Obstetrics (FIGO) staging system of cervical cancer, the treatment approach and prognosis depend on tumor size and tumor stage, and the treatment is primarily by surgery or radiation therapy with chemotherapy a valuable adjunct in recent years [2, 3]. However, these can lead to many serious complications, including pelvic adhesions, intrapelvic nerves injuries leading impairment of urination, defecation, and sexual function, and consequent deterioration of postoperative quality of life (QOL), as well as the yearly increasing incidence of rectal cancers after pelvic radiation [4, 5]. Even the early stage of invasive cervical cancer in FIGO Stage IB1 also need radical hysterectomy with pelvic lymphadenectomy, still with 7.3% recurrences and 4.6% mortality [6]. For microinvasive cervical cancer, patients in FIGO Stage IA (stromal invasion depth < 5 mm) are treated primarily with cervical conization or total extrafascial hysterectomy with less injury [7–10], and are associated with a better prognosis and almost no recurrence [3, 6]. Hence, it is of great importance that detecting and diagnosing microinvasive cervical cancer to improve prognosis, reduce recurrence and mortality.

The current diagnostic methods of microinvasive cervical cancer lesions are mainly imaging diagnosis and pathological evaluation [3]. Pathological evaluation is the gold standard but obtained through invasive surgery (a loop electrosurgical excision procedure or cone biopsy). Imaging plays a central role in the most recent FIGO 2018 classification for cervical cancer, including ultrasound, magnetic resonance imaging (MRI) and computed tomography (CT) [11, 12]. They have high accuracy for measurement of primary tumor size in invasive cervical cancer [11, 12]. Among them, ultrasound is comparable, or even superior to MRI, in detecting small cervical cancers (≤ 1 cm^3^) [13] and early-stage cervical cancer (FIGO IA2-IIA) [14]. In recent years, contrast-enhanced ultrasound (CEUS) is a powerful technique that can augment the intensity of tumor microvascular (diameters as small as 40 μm) flow signals by using ultrasound microbubbles to increase the signal-to-noise ratio [15–18], and can improve the diagnostic performance of tumor lesions accordingly [19, 20]. However, extremely low diagnostic performance for microinvasive cervical cancer [21–23]. Therefore, there is a pressing demand for a more accurate and noninvasive imaging modality to detect microinvasive cervical cancer (FIGO Stage IA).

Based on CEUS, with the application of targeted microbubbles, a novel ultrasound molecular imaging (USMI) is more appealing and sensitive modality for the qualitative or quantitative evaluation of pathophysiological changes at the molecular level to increase the detection of extremely early tumors in recent years [24]. Tumor-associated microvessel networks are enriched in the onset of tumor growth [25, 26], so markers targeting to tumor angiogenesis could improve diagnostic efficiency. And the vascular endothelial growth factor receptor type 2 (VEGFR2) is highly up-regulated during the onset of tumor angiogenesis, overexpresses at tumor sites [27, 28]. Therefore, it is the more common target among angiogenesis markers [17]. MB_VEGFR2_-based USMI has been applied for the early detection of pancreatic ductal adenocarcinoma [29], breast cancer and ductal carcinoma in situ (DCIS) [30, 31], and hepatic micrometastases [32]. Nonetheless, it remains unknown whether MB_VEGFR2_-based USMI can increase the detection of microinvasive cervical cancer to improve the prognosis of this patient population.

**Fig. 1:**
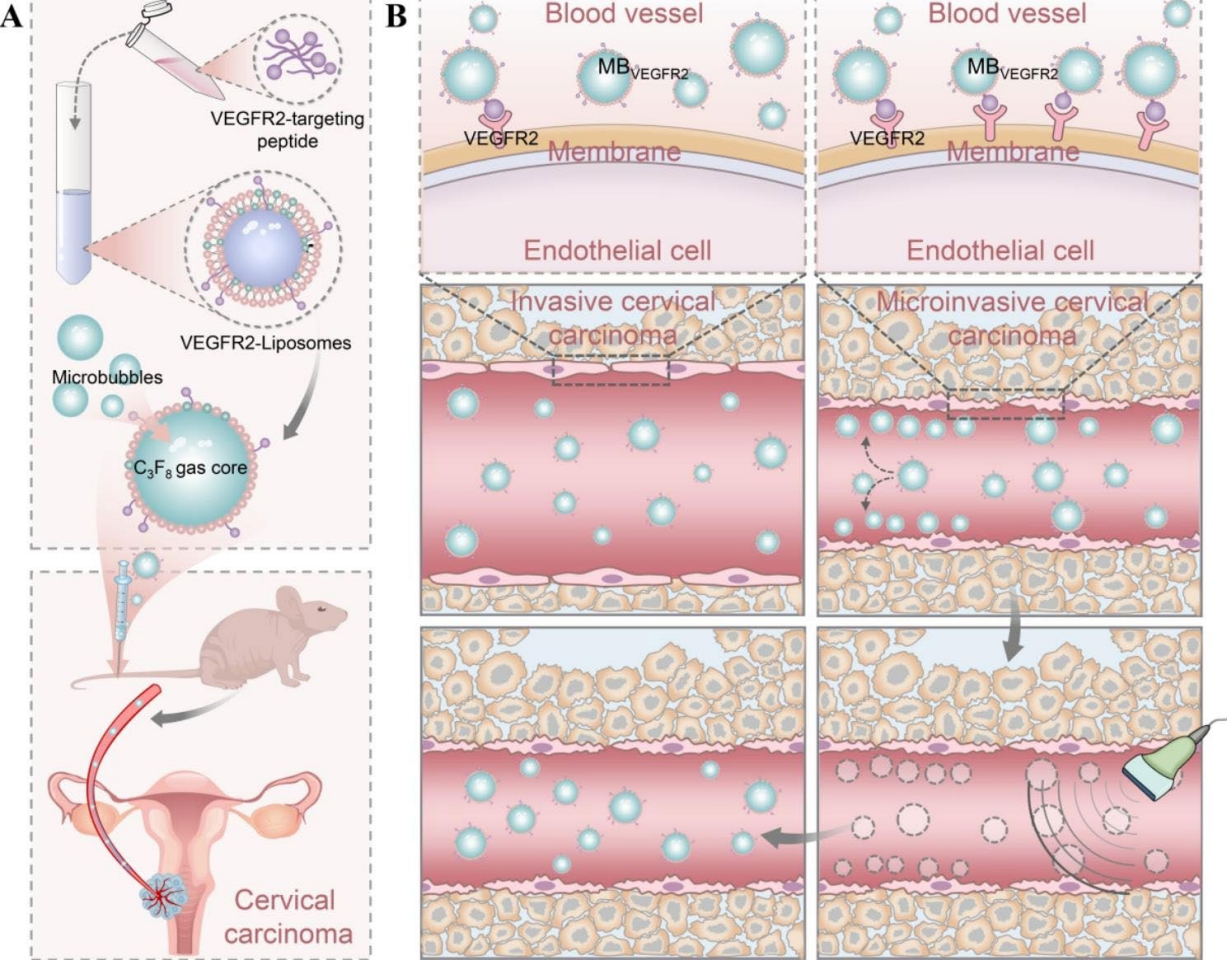
Schematic illustration of the preparation of MB_VEGFR2_ and MB_VEGFR2_-based molecular ultrasound imaging for earlier detection of microinvasive cervical cancer in vivo. (**A**) Synthesis of MB_VEGFR2_. (**B**) Molecular ultrasound imaging of microinvasive cervical cancer via MB_VEGFR2_. After cervical cancer modeling in mice, MB_VEGFR2_ suspension was injected into the tail vein. Based on the clinical ultrasound imaging system, the high frequency linear probe was used to achieve the VEGFR2-targeted molecular ultrasound imaging by means of destructive replenishment. The contrast signals of the adherent microbubbles and circulating microbubbles were continuously captured for 30 s. Then all the microbubbles were destroyed using a destructive pulse with a high mechanical index for 1 s. Ultrasound imaging was performed for 10 s to obtain the signal of freely circulating microbubbles after destruction. Thus, the quantification of targeted signals was calculated by the difference between the signal intensity before and after destruction, which was represented as the normalized intensity difference (NID). Perfluoropropane gas, C_3_F_8_; Vascular endothelial growth factor receptor type 2 (VEGFR2) targeted microbubble, MB_VEGFR2_;

Herein, we constructed a novel VEGFR2-targeted microbubble (MB_VEGFR2_, Fig. 1) to explore the feasibility of earlier detection of microinvasive cervical cancer in mice models to improve the imaging diagnosis of microinvasive cervical cancer (FIGO Stage IA). MB_VEGFR2_ was prepared using maleimide-thiol conjugation; the targeting ligands were directly incorporated into the microbubble shell to facilitate clinical translation. Animal studies were performed to assess the value of this noninvasive imaging method as a screening tool for microinvasive cervical cancer, given that Stage IA diagnoses are currently primarily based on microscopic examination.

## Results

### Characterization of microbubbles

**Fig. 2:**
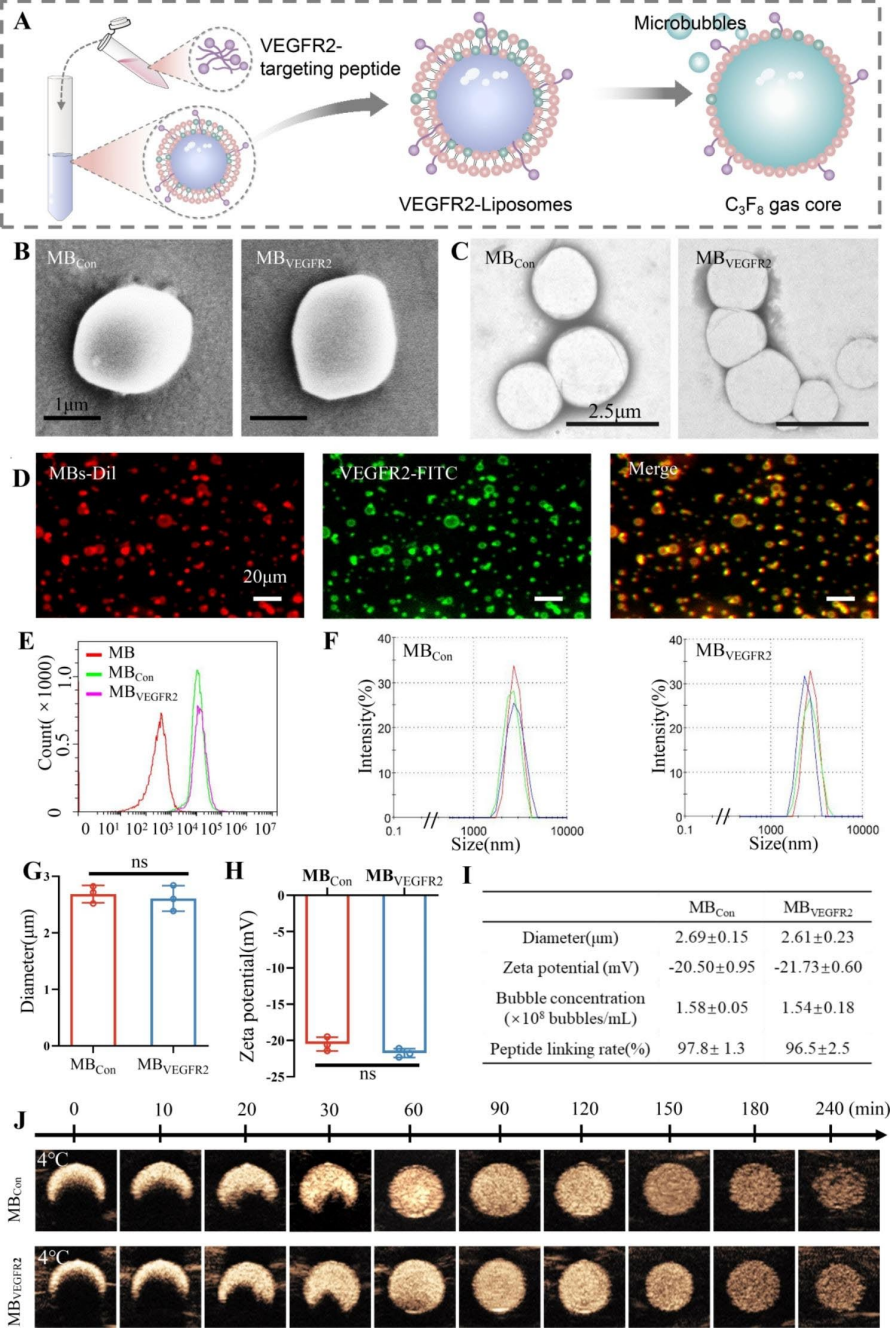
Characterization of microbubbles (MBs). (**A**) Synthesis of MB_VEGFR2_. (**B**) Observation of morphology and structure of MB_VEGFR2_ by scanning electron microscopy (SEM) images: MB_Con_ versus MB_VEGFR2_; scale = 1 μm. (**C**) Observation by transmission electron microscopy (TEM) images: MB_Con_ versus MB_VEGFR2_; scale = 2.5 μm. (**D**) Observation of MB_VEGFR2_ by fluorescence microscopy (400 ×; scale bar, 20 μm). The lipid shells of microbubbles were stained with red fluorescent dye DiI. FITC-labeled secondary antibodies were used to trace VEGFR2. (**E**) Flow cytometry analysis of peptide-linking rate of MB_Con_ and MB_VEGFR2_. **(F**) Size distribution by the intensity of MB_Con_ and MB_VEGFR2_. (**G**) Box-and-whisker plot of mean diameters of MB_Con_ and MB_VEGFR2_. (**H**) Box-and-whisker plot of mean zeta potentials of MB_Con_ and MB_VEGFR2_. (**I**) Detailed display about mean diameters, zeta potentials, bubble concentrations and peptide linking rate of MB_Con_ and MB_VEGFR2_. (**J**) The stability and imaging ability of MB_Con_ and MB_VEGFR2_ at 4℃ were determined by micro ultrasound system using an agarose mold. (ns = nonsignificant)

Preparation procedure of MB_VEGFR2_ was shown in Fig. 2A, The VEGFR2-targeting peptide was hybridized with the liposomes and then the gas C_3_F_8_ was exchanged to obtain MB_VEGFR2_. The morphology of MB_Con_ and MB_VEGFR2_ were determined by scanning electron microscopy (SEM) and transmission electron microscopy (TEM). As shown in Fig. 2B C, MB_Con_ and MB_VEGFR2_ presented spherical shapes with similar sizes. The irregular surfaces may be ascribed to the peptides incorporated into MB shells.

A fluorescence assay was conducted to validate the successful integration of VEGFR2-targeting peptide S1 with liposomes in MB_VEGFR2_. The red and green fluorescence signals almost overlapped, demonstrating the successful binding of VEGFR2-targeting peptide S1 to the microbubbles (Fig. 2D).

Moreover, flow cytometry analysis showed the peptide-linking rate of MB_VEGFR2_ was comparable to MB_Con_ (96.5 ± 2.5% vs. 97.8 ± 1.3%; *P =* 0.467; Fig. 2E and I), which further demonstrated that VEGFR2-targeting peptide S1 and nontargeting peptide efficiently combined with the microbubbles.

As shown in Fig. 2F, G and I, dynamic light scattering imaging showed that the mean diameter of MB_VEGFR2_ and MB_Con_ were comparable (2.61 ± 0.23 μm vs. 2.69 ± 0.15 μm, *P* = 0.65). In addition, MB_VEGFR2_ showed a zeta potential similar to MB_Con_ (-21.73 ± 0.60 mV vs. -20.50 ± 0.95 mV; *P =* 0.13; Fig. 2H and I).

As shown in Fig. 2J, the US signal intensities were stable for up to 120 min in both MB_Con_ and MB_VEGFR2_ at 4℃. After 120 min, the signal intensities of both MBs began to decrease gradually. Moreover, with increasing temperature, the US signal intensities at 37℃ were slightly worse than at 4℃ in both MB_Con_ and MB_VEGFR2_, which were stable for up to 90 min and decreased gradually (Figure S2).

The Fourier-transform infrared (FTIR) spectrum of liposomes showed a broad N-H stretch band at 3200–3600 cm^− 1^ and a weak C = O stretching vibrational absorption at 1660 cm^− 1^. In the spectrum of VEGFR2-liposomes, due to the existence of more secondary amide groups in the structure of VEGFR2-targeting peptide, the intensity of the two bands remarkably increased. These results demonstrated the successful synthesis of VEGFR2-targeting peptide and liposomes, as showed in Figure S3.

### In vitro adhesion ability and biocompatibility of MB_VEGFR2_

**Fig. 3:**
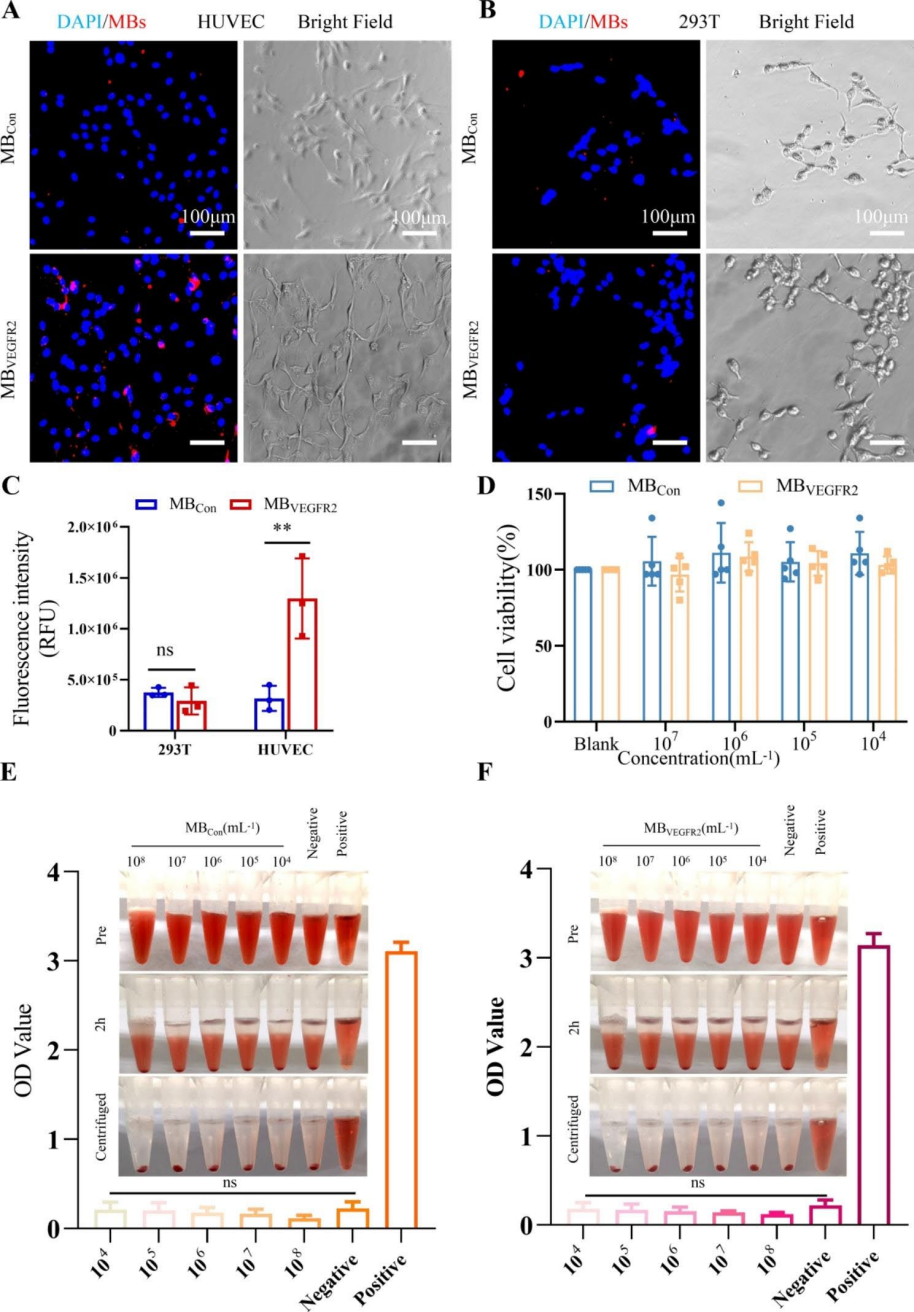
The adhesion ability and biocompatibility of MB_Con_ and MB_VEGFR2_ in vitro. (**A**) Specific attachment of MB_Con_ or MB_VEGFR2_ to human umbilical vein endothelial cells (HUVECs) with high expression of VEGFR2 was observed at inverted fluorescence microscopy. (**B**) Specific attachment of MB_Con_ or MB_VEGFR2_ to human embryonic kidney cell line 293T with VEGFR2-nonexpression was observed at inverted fluorescence microscopy. (× 200; scale bar, 25 μm). (**C**) Quantification analysis of MB_Con,_ MB_VEGFR2_ attachment to HUVECs and 293T respectively. (**D**) The cytocompatibility of different concentrations of MB_Con_ and MB_VEGFR2_ on HUVECs was determined using CCK8 method. Hemolysis of red blood cells in the presence of MB_Con_(**E**) and MB_VEGFR2_(**F**) with various concentrations (n = 4). PBS and distilled water were used as negative and positive controls, respectively. (** *P* < 0.01, ns = nonsignificant)

Fluorescence and bright field imaging demonstrated that MB_VEGFR2_ yielded significantly stronger attachment of HUVECs (VEGFR2-high expression) cells (Fig. 3A) compared with MB_Con_; The fluorescence intensity levels in MB_VEGFR2_ groups were about 3-fold higher than in MB_Con_ groups (Fig. 3C). However, both MBs showed no effective attachment after incubation with 293T (VEGFR2-nonexpression) cells (Fig. 3B). Moreover, there was no significant difference in fluorescence intensity levels between cells incubated with MB_Con_ and MB_VEGFR2_ (*P* = 0.361, Fig. 3C).

Cell cytotoxicity and hemolysis tests were conducted to measure the biocompatibility of MB_VEGFR2_ in vitro. HUVECs were cultured with MB_Con_ and MB_VEGFR2_. Compared with the blank group, different concentrations of MB_Con_ and MB_VEGFR2_ had no obvious influence on cell viability after incubation. There was no significant difference in cell viability in MB_Con_ (*P* = 0.717) and MB_VEGFR2_ (*P* = 0.220) (Fig. 3D). The hemolysis test results are shown in Fig. 3E and F. Hemolysis was conducted in distilled water as a positive control. Like PBS (negative control), there was no hemolysis or agglutination of erythrocytes with different concentrations of MB_Con_ (Fig. 3E) and MB_VEGFR2_ (Fig. 3F). Besides, the supernatants were transparent and colorless after centrifugation. Moreover, there was no significant difference in OD values of supernatants between PBS and MB_Con_ (*P* = 0.280), PBS and MB_VEGFR2_ (*P* = 0.129), which suggested they were non-toxic.

### Pathological changes and microvessel density in cervical cancer mice

**Fig. 4:**
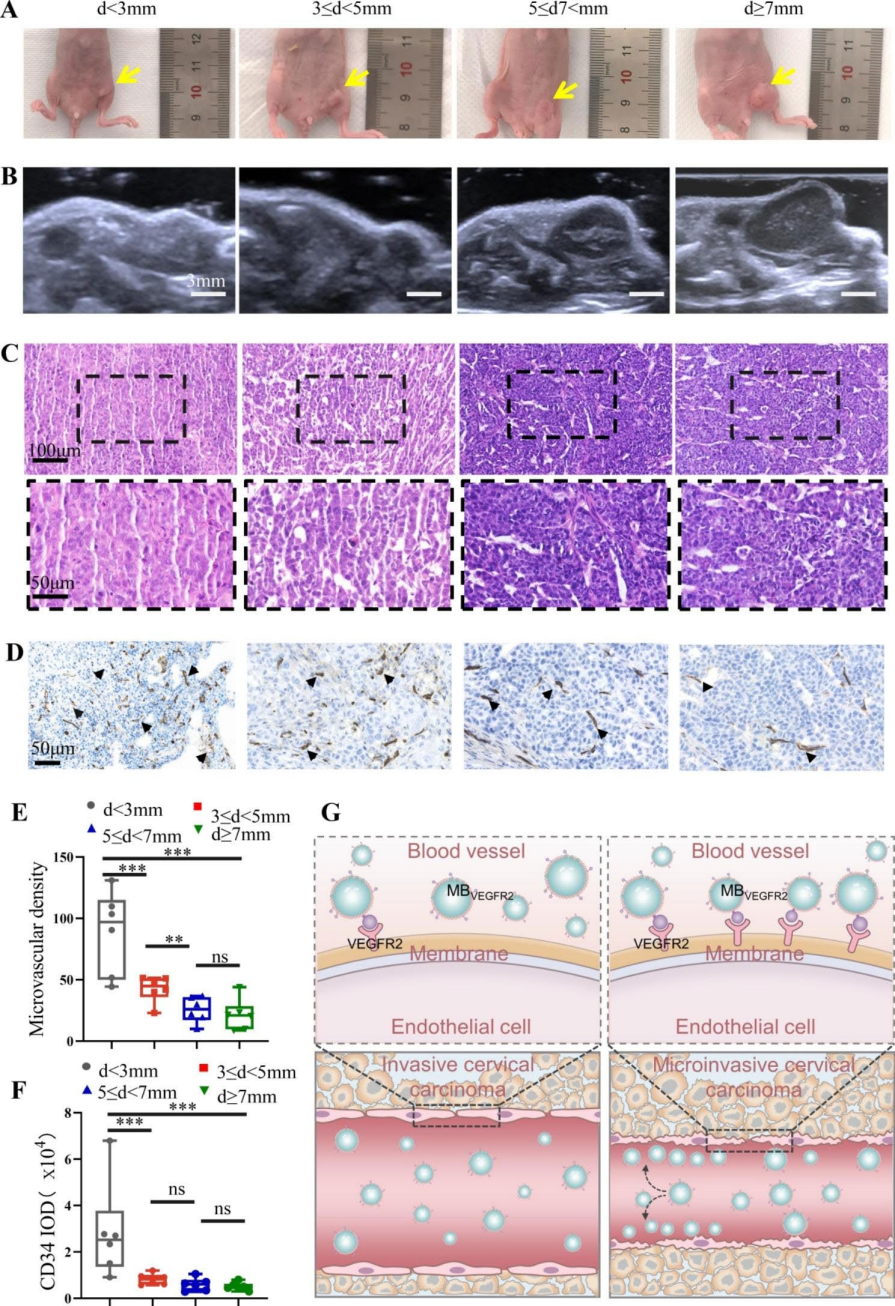
Histopathologic changes in different diameters of cervical cancer. (**A**) Representative images of different diameters xenograft groups. (**B**) Representative ultrasound images of different diameters xenograft groups. (**C**) Pathological images of H&E staining (× 200; scale bar, 100 μm) of different groups. (**D**) Immunohistochemical staining to evaluate CD34-microvessel density (MVD) in different groups (× 400; scale bar, 50 μm). (**E**) Box-and-whisker plot of CD34-MVD in different diameters of cervical cancer. (**F**) Box-and-whisker plot of CD34-IOD in different diameters of cervical cancer. (** *P* < 0.01, *** *P* < 0.001, ns = nonsignificant). (**G**) MVD which revealed the tumor neovascularization process, was more abundant in microinvasive cervical cancer models then invasive cervical cancer. MB_VEGFR2_ could targetly bind to angiogenesis markers-the vascular endothelial growth factor receptor type 2 (VEGFR2) on the new microvessels

Based on the criteria for FIGO Stage IA1 (stromal invasion < 3 mm in depth), IA2 (stromal invasion ≥ 3 mm and < 5 mm in depth) and greater, the mice were.

divided into 4 groups (diameter < 3 mm, 3–5 mm, 5–7 mm, and ≥ 7 mm) (Fig. 4A), and the diameter was measured with US (Fig. 4B). Tumor cells were observed in each mouse on H&E staining (Fig. 4C), which indicated the success of our early cervical cancer model. Staining of tumor tissue for CD34-MVD revealed the tumor neovascularization process. As shown in Fig. 4D and E, significant angiogenesis was observed in the very early tumors (d < 3 mm: 88.53 ± 6.33); MVD decreased with increasing tumor sizes (3 ≤ d < 5 mm: 42.67 ± 3.03; 5 ≤ d < 7 mm: 25.70 ± 2.11; d ≥ 7 mm: 21.40 ± 2.29, all *P <* 0.001). In addition, the MVD of the 3 ≤ d < 5 mm group decreased significantly compared with the d < 3 mm group (*P <* 0.001, about 2-fold), and the MVD in the 5 ≤ d < 7 mm group decreased significantly compared with the 3 ≤ d < 5 mm group (*P <* 0.01, about 1.7-fold). Furthermore, as shown in Fig. 4F, the highest expression of CD34 was in the d < 3 mm group, CD34 decreased gradually along with increasing diameter of tumors (all *P* < 0.001). And it was higher in d < 3 mm group than in the 3 ≤ d < 5 mm group (*P* < 0.001). These results further demonstrated that the expression of angiogenesis markers was the most abundant in microinvasive cervical cancer models (Fig. 4G).

### In vivo superb microvascular imaging of cervical cancer

**Fig. 5:**
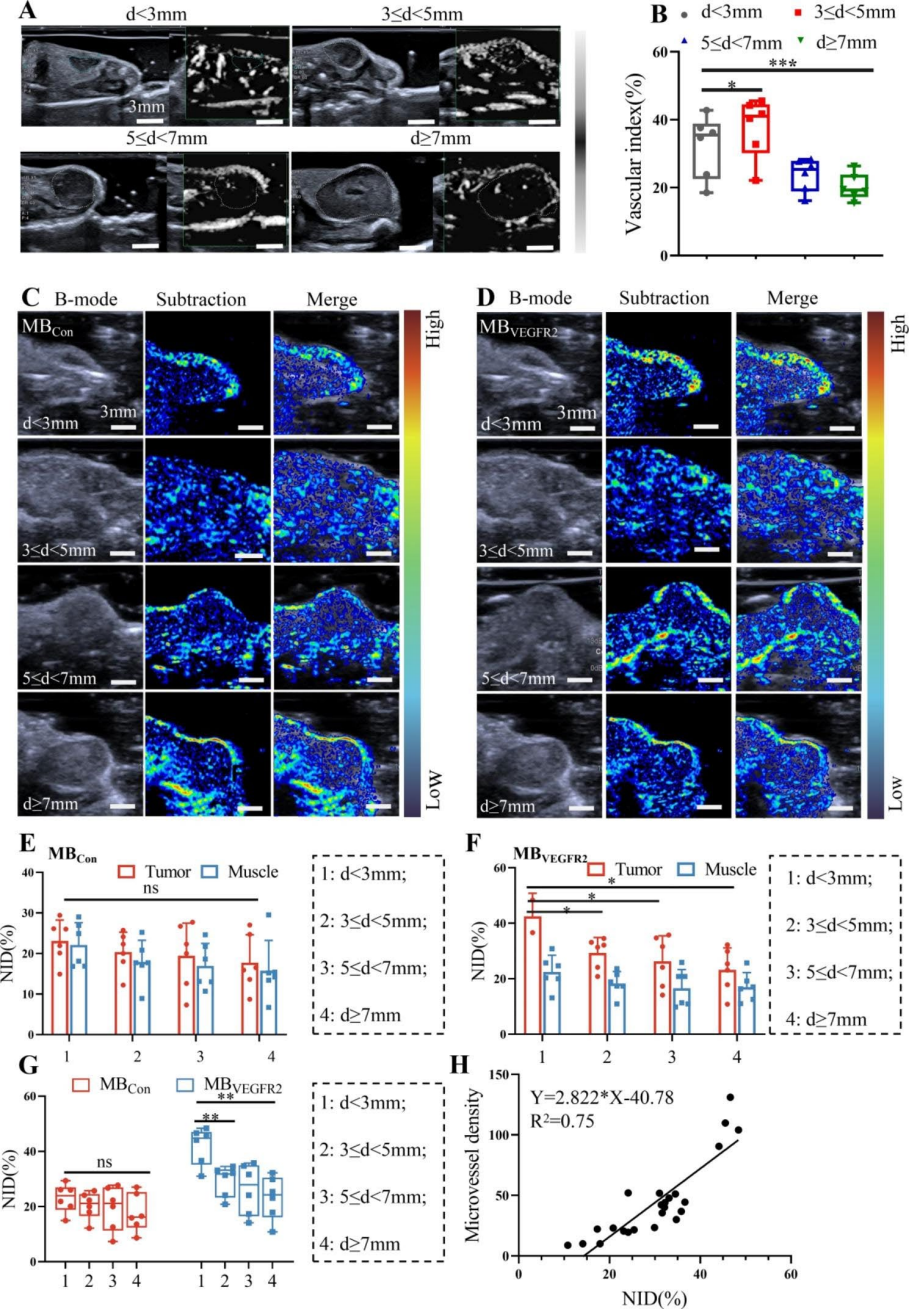
Representative images of US molecular imaging USMI in identifying cervical cancer. (**A**) Representative images of superb microvascular imaging (SMI) in different diameters of cervical cancer. (**B**) Box-and-whisker plot of quantitative parameter of SMI in different diameters. Representative ultrasound images in B-mode (gray) and molecular ultrasound images (color coded) after administrations of MB_Con_(**C**) and MB_VEGFR2_(**D**). (**E**) Quantitative analyses of USMI signal intensity about MB_Con_ in tumor and muscle tissues of each group. (**F**) Quantitative analyses of USMI signal intensity about MB_VEGFR2_ in tumor and muscle tissues of each group. (**G**) Box-and-whisker plot of USMI signal intensity on the basis of NID in different diameters of cervical cancer with MB_Con_ or MB_VEGFR2_ administration. (**H**) Linear correlation analysis between the NID and CD34-MVD. (* *P* < 0.05, ** *P* < 0.01, ns = nonsignificant)

Given that SMI is sensitive in detecting low-velocity blood flow, it was used to visualize the blood supply of cervical cancer with different sizes. For SMI imaging in Fig. 5A and B, the 3 ≤ d < 5 mm group exhibited the highest vascular index (37.79 ± 2.17), and the value of VI significantly decreased along with an increasing tumor diameter (5 ≤ d < 7 mm: 23.78 ± 1.28, d ≥ 7 mm: 20.19 ± 1.05, all *P* < 0.001). The tumors in the d < 3 mm group also had a significantly lower vascular index (32.33 ± 2.31, *P* < 0.05) than the 3 ≤ d < 5 mm group, which was inconsistent with pathological CD34-MVD. These results indicated the heterogeneity in microvascular perfusion for cervical cancer of different sizes.

### Identifying microinvasive cervical cancer by MB_VEGFR2_-based USMI

To explore whether MB_VEGFR2_-based USMI can be applied to distinguish microinvasive (FIGO Stage IA) from normal tissue, USMI was performed for each mouse. When mice were injected with MB_Con_, all tumors could not be identified from surrounding muscles in subtracted ultrasound images (Fig. 5C and E, all *P* = 0.891). On the other hand, we found a significant contrast enhancement between tumors and surrounding muscles after MB_VEGFR2_ administration (Fig. 5D F, all *P* < 0.05), which clearly defined the tumor margin from surrounding muscles and revealed the in vivo tumor-specificity of MB_VEGFR2_. The very early tumors (d < 3 mm) exhibited maximum contrast enhancement (NID of tumor: 42.03 ± 2.76% vs. NID of muscles: 22.43 ± 2.47%, about 1.87-fold) in subtracted ultrasound images. The contrast signal intensity gradually decreased as the tumor size increased (about 1.60-fold in 3 ≤ d < 5 mm; 1.58-fold in 5 ≤ d < 7 mm, and 1.37-fold in d ≥ 7 mm, all *P* < 0.05).

Furthermore, we found the signal intensity gradually decreased after MB_VEGFR2_ administration as the tumor increased. The NID values in very small tumors (d < 3 mm: 42.03 ± 2.76%) showed significantly higher signal intensity after MB_VEGFR2_ administration with increasing tumor sizes (3 ≤ d < 5 mm: 29.33 ± 2.26%; 5 ≤ d < 7 mm: 26.27 ± 3.76%; d ≥ 7 mm: 23.22 ± 3.23%; all *P* < 0.01) (Fig. 5G). Differences in NID values were also observed between d < 3 mm and 3 ≤ d < 5 mm (*P* < 0.01). In addition, there was no evidence of differences in NID values when mice were administered MB_Con_ (d < 3 mm: 23.07 ± 2.12%; 3 ≤ d < 5 mm: 20.38 ± 1.99%; 5 ≤ d < 7 mm: 19.43 ± 3.28%; d ≥ 7 mm: 17.73 ± 2.82%; all *P* = 0.545). Moreover, we identified a good linear correlation (R^2^ = 0.75, *P* < 0.0001) between the NID values and CD34-MVD (Fig. 5H). Overall, these results preliminarily demonstrated that MB_VEGFR2_-based USMI could assist in distinguishing microinvasive cervical cancer from normal tissues and could help identify lesions undetected by MRI or conventional ultrasound during clinical practice.

### Safety of MB_VEGFR2_ in vivo

**Fig. 6:**
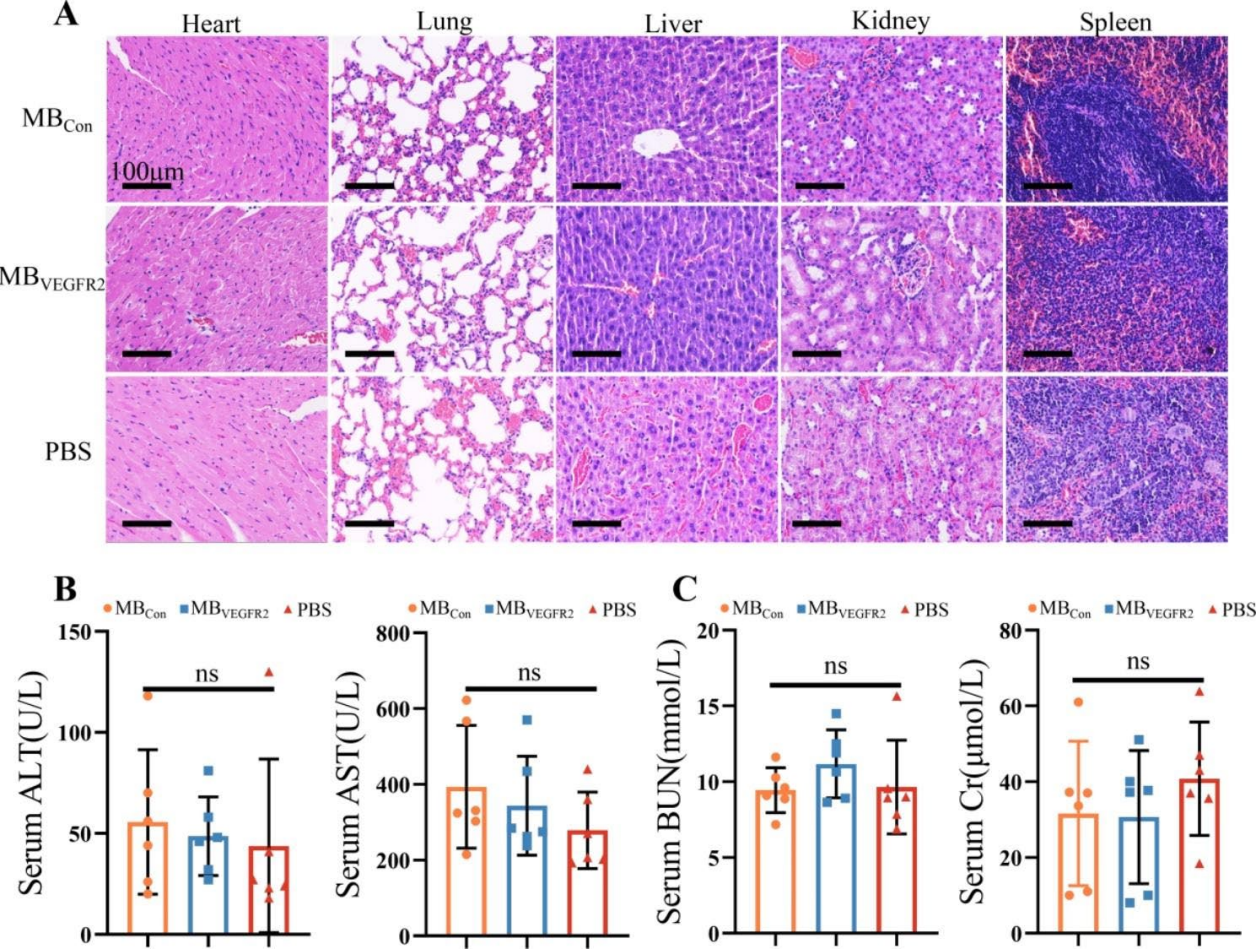
Biocompatibility of MBs in vivo. (**A**) One day after injection of 100 times the conventional dose of MB_Con,_ MB_VEGFR2_ and PBS, representative histopathologic images of major organs (from left to right: heart, lung, liver, kidney, spleen) with H&E staining. (**B**) and (**C**) One day after injection of 100 times the conventional dose of MB_Con,_ MB_VEGFR2_ and PBS, serum biochemical values, including alanine aminotransferase (ALT), aspartate aminotransferase (AST), blood urea nitrogen (BUN) and creatinine (Cr). (ns = nonsignificant)

As for the safety of MB_VEGFR2_ administered in vivo, there was no significant difference in pathological abnormalities or damage after administration of MB_Con_, MB_VEGFR2_ or PBS, based on H&E staining of the major organs (heart, lung, liver, kidney, spleen) (Fig. 6A). In addition, no significant differences were observed in serum biomarkers (AST, ALT, creatinine, blood urea nitrogen) after injection of MB_Con_, MB_VEGFR2_ or PBS (Fig. 6B C) one day later.

## Discussion

The sub-staging about tumor size in FIGO staging classification was based on the increasing evidences that tumor size provides an evident prognostic value. However, early imaging diagnosis of microinvasive cervical carcinoma (Stage IA1, IA2) is often challenging. Our study demonstrated that cervical carcinoma less than 3 mm could be distinguished from surrounding normal tissues by US molecular imaging with VEGFR2. Moreover, USMI with VEGFR2 was more sensitive to detecting extremely early tumors than larger ones. This easy-to-perform, noninvasive imaging method may be a potent monitoring tool for microinvasive cervical carcinoma.

Imaging assessments have been incorporated in the revised 2018 FIGO staging system for cervical cancer [3]. Applications of imaging techniques for the pretreatment evaluation of cervical cancer have been increasing. However, MRI has long been considered the most promising technique in the primary evaluation of patients with cervical cancers [33]. Ultrasound has recently gained popularity with the advantage of low costs, faster examination time, and wide availability [34]. It has been reported that MRI and pelvic ultrasound’s accuracy in assessing tumor size in small versus large tumors are comparable [35]. Therefore, ultrasound is an accurate preoperative staging imaging technique complementary to MRI in women with early-stage cervical cancer, especially in lower-resource settings. However, early imaging diagnosis of microinvasive cervical carcinoma (Stage IA1, IA2) is difficult during clinical practice since these imaging modalities reflect structural change rather than early molecular change. Thus, developing a potent imaging tool enabling molecular and cellular visualization is essential for detecting microinvasive cervical carcinoma. Molecular imaging has emerged as an ideal technique to achieve such a goal. Among various imaging modalities, ultrasound has huge prospects for application in microinvasive cervical carcinoma monitoring due to its advantages, including convenience, absence of ionic radiation and cost-effectiveness. These provide the basis to employ USMI rather than MRI/PET molecular imaging for microinvasive cervical carcinoma evaluation.

MB_VEGFR2_-based USMI has been demonstrated to be a highly sensitive and suitable technique for distinguishing angiogenesis and vascularisation in solid tumors of a well-discernible size [36, 37]. However, to our knowledge, its potential in monitoring microinvasive cervical carcinoma (Stage IA1, IA2) has not been analyzed so far. Interestingly, we found cervical carcinoma less than 3 mm could be distinguished by USMI with VEGFR2 (*P* < 0.05), and the sensitivity gradually decreased as the tumor size increased. Moreover, the MB_VEGFR2_-based USMI signal matched well with histologic MVD in cervical carcinoma lesions (R^2^ = 0.75, *P* < 0.0001). The highest NID was detected in lesions less than 3 mm, whereas the NID levels significantly decreased with increasing tumor size. Angiogenesis may occur in very small, proliferating tumors [38], leading to significant upregulation of VEGFR2 and a strong angiogenic response [39]. With ongoing tumor growth, VEGFR2 expression is gradually downregulated [31]. USMI with MB_VEGFR2_ in our study allows the assessment of tumor angiogenesis expression in cervical cancer mice. Accordingly, it has huge prospects for the noninvasive assessment of microinvasive cervical carcinoma (Stage IA1, IA2) patients.

Besides, VEGFR2-targeted microbubbles in our study were prepared using maleimide-thiol conjugation, not by the biotin-streptavidin bridging chemistry method. It is widely acknowledged that streptavidin is immunogenic and can cause severe allergic reactions in patients [40, 41]. These first-generation targeted contrast agents based USMI in patients has not been translated to clinical practice. Novel VEGFR2-targeted microbubbles-BR55 have been developed and are lipopeptide-based without potentially immunogenic proteins [36, 37]. The targeting ligands are directly incorporated into the microbubble shell in BR55, making it potentially suitable for clinical translation. Thence, MB_VEGFR2_ in our study using maleimide-thiol conjugation was successfully synthesized, similar to BR55. Current evidence suggests that BR55 has great potential for improving earlier detection of pancreatic ductal adenocarcinoma [29], breast cancer and ductal carcinoma in situ (DCIS) [30, 31], and hepatic metastases of colon cancer [32]. USMI with MB_VEGFR2_ has hitherto not been applied for the early detection of cervical carcinoma (Stage IA1, IA2). Therefore, our study lays the groundwork for applying this promising imaging approach for early microinvasive cervical carcinoma (Stage IA1, IA2) detection.

The safety of MB_Con_ and MB_VEGFR2_ in vitro and in vivo has also been demonstrated in the present study, as all components of the mentioned MBs were highly biocompatible. MB_VEGFR2_ functioned as a polypeptide-functionalized microbubble in our study. Based on this design, no side effects were observed in mice that received a dual intravenous injection.

Nevertheless, the following limitations of our study need to be addressed. First, it should be borne in mind that clinical ultrasound devices for the cervix (endovaginal or transabdominal transducers) have a slightly lower resolution than the high-frequency ultrasound system used in the present study. Moreover, compared with mice, some differences may affect the performance of USMI in humans, including imaging depth and so on. Another limitation is the use of only one cervical cancer model. It should be borne in mind that the onset of angiogenesis and the early angiogenic response of other cervical cancer models may differ from the Hela model and should thus be further elucidated. Indeed, further studies in other cervical cancer models or humans are required to confirm the potential of USMI with VEGFR2 for early and accurate assessment.

## Conclusion

In this study, our results substantiate that MB_VEGFR2_-based USMI has huge prospects for monitoring high angiogenic activity in microinvasive cervical cancer, suggesting its potential for detecting and characterizing these extremely early lesions. This study provides an important foundation for the clinical translation of a molecularly-targeted US-based screening modality for detecting microinvasive cervical carcinoma (Stage IA1, IA2).

## Materials and methods

### Cervical cancer xenograft

The Hela cervical cancer cell line was purchased from the China Center for Type Culture Collection (CCTCC, Wuhan, China). They were grown in DMEM (GIBCO) supplemented with 10% fetal bovine serum (FBS, GIBCO) and 1% penicillin/streptomycin (Thermo Fisher Scientific) in a 5% CO_2_ humidified incubator at 37 °C.

SPF-level female BALB/c nude mice (4–6 weeks old; 18–22 g) were obtained from Guangdong GemPharmatech Co., Ltd and maintained under standard environmental conditions. All protocols involving animals were approved by the local ethics committee of the Animal Care Committee of South China Agricultural University (2022d075).

To establish tumor models, we subcutaneously injected 1 × 10^7^ Hela cells, suspended in 100 μL PBS per mouse, into the right groin area. And the diameter of tumor was measured with B-mode ultrasound by using electronic calipers every two days. Tumor volumes were determined using the formula 1/6π × width^2^ × length. To determine whether MB_VEGFR2_-based USMI can diagnose microinvasive cervical cancer, the mice were expected to be divided into 4 groups (the diameter of tumors < 3 mm, 3–5 mm, 5–7 mm, and ≥ 7 mm, n = 6 in each group) based on the criteria for FIGO Stage IA1 (stromal invasion < 3 mm in depth), IA2 (stromal invasion ≥ 3 mm and < 5 mm in depth) and greater. When the diameter of tumor met the requirements for grouping, mice were randomly assigned to each group for subsequent experiments.

### Preparation and characterization of microbubbles

MB_VEGFR2_ used in our study was based on maleimide-thiol conjugation; the targeting VEGFR2 ligands were directly incorporated into the microbubble shell. The VEGFR2-targeting peptide S1 (H_2_N-Leu-Ile-Asn-His-Glu-Trp-Lys-Asn-Tyr-Phe-Pro.

-Leu-Ser-Phe-COOH) has previously been reported in the literature [42, 43]. Cysteine was introduced at the C-terminal of S1 (S1C, LIDHEWKENYFPLSFC) to provide a thiol group. Briefly, all phospholipids (18 mg of DPPC, 3.5 mg of DSPE-PEG_2000_-MAL, 1 mg of DPPA) were dissolved in 4 mL chloroform and evaporated at 60℃ for 30 min by a rotary evaporator to form a thin phospholipid film. Then the membrane was hydrated with 8 mL PBS (with 10% glycerol (v/v) and 2 mg/mL Pluronic F-68) at 60℃ and 120 rpm in a rotary evaporator for 60 min to form the maleimide-liposome. Afterward, S1 was mixed with the maleimide-liposome with a - HS: -maleimide molar ratio of 1:1 and incubated at 4℃ overnight to prepare the S1-maleimide-liposome. Subsequently, the air above the liquid was exchanged with 10 mL of perfluoropropane (C_3_F_8_), and a shaker was used for the mechanical vibration of the liposome solution for 45 s to form MBs. Finally, the suspension was centrifuged (805 ×g, 5 min) three times and resuspended in PBS. We referred to S1-MBs as MB_VEGFR2_ in the rest of the manuscript. The nontargeted peptide (isotype control)-MBs were prepared similarly and called MB_Con_.

SEM and TEM were used to observe the morphology and structure of MB_Con_ and MB_VEGFR2_. The processed specimens were viewed and photographed by a Merlin scanning electron microscope (Carl Zeiss, Germany) and Tecnai G2 Spirit electron microscope (FEI, USA), respectively. The samples for TEM were negatively stained with a 2% phosphotungstic acid solution.

Fluorescence microscopy (Nikon, Tokyo, Japan) was used to determine the specific binding of VEGFR2 to the surface of MBs. DiI was added to fluorescently labeled MBs before liposomes were formed, and FITC-labeled secondary antibodies (Cell Signaling Technology, 2 mg/mL) were used to trace VEGFR2. VEGFR2-FITC-MBs were synthesized to calculate the S1 peptide-linking rate. VEGFR2-FITC-MB solution was diluted in PBS (1:500 v/v) and analyzed using a flow cytometer at FITC. Three repeated measurements for each sample were performed. The structures of the final products (liposomes, VEGFR2-liposomes) were characterized by FTIR spectroscopy (Nicolet 6700, Thermo Fisher, USA) to further determine the modification of maleimide-thiol conjugation.

Particle sizes and zeta potentials were evaluated by dynamic light scattering (DLS) measurement (Malvern Zetasizer Nano, Malvern, U.K.) at 25℃; each sample underwent three measurements.

The bubble concentration was finally calculated with an Automated Cell Counter (Bio-Rad, USA). Briefly, 10 μL of MB_VEGFR2_ or MB_Con_ diluted 20 times in PBS was added to counting slides, then the concentration of MBs was calculated automatically. The experiment was repeated three times.

The in vitro stability of MB_VEGFR2_ and MB_Con_ were compared using a custom-made 2% (w/v) agarose mold as previously described at 4℃ and 37℃ respectively [44]. 1 mL of MBs in PBS (10^7^ MB/mL concentration) was added to the sample well. A clinical US scanner (Acuson Sequoia ultrasound system, Siemens, Erlangen, Germany) with a 10L4 high-frequency (2.9–9.9 MHz) probe was used in contrast pulse sequencing mode with the following imaging parameters: Low frequency, a gain of 0 dB, an image depth of 4 cm, an acoustic output of 0.2%, a dynamic range of 70 dB, and MI of 0.09. The focal area was placed at the center of the sample well. The horizontal imaging plane through the agarose mold was used.

### In vitro specific targeting ability of MB_VEGFR2_

Human umbilical vein endothelial cells (HUVECs, ScienCell) with high expression of VEGFR2 were cultured in endothelial cell medium (ECM, ScienCell) containing 5% FBS, 1% endothelial cell growth supplement and 1% penicillin/streptomycin in a 5% CO_2_ humidified incubator at 37℃. The human embryonic kidney cell line 293T (VEGFR2-nonexpression) was used as a control cell model and cultured in DMEM (GIBCO) supplemented with 10% FBS (GIBCO) and 1% penicillin/streptomycin (Thermo Fisher Scientific) at 37℃ in a humidified atmosphere with 5% CO_2_. The HUVECs and 293T cells were seeded in 12-well plates at a density of 1 × 10^5^ cells/well and incubated overnight. MB_VEGFR2_ and MB_Con_ were both traced by fluorescent dye DiI during preparation. The next day, the culture media were discarded; the cells were washed with 0.5 mL PBS and then fixed with 0.5 mL methanol for 15 min at 37℃. After washing, the cells were incubated in 0.5 mL PBS containing 2 × 10^7^ MB_VEGFR2_ or MB_Con_ at 37℃ for 30 min in a shaking incubator. Finally, all cells were washed with PBS three times, stained with DAPI (Beyotime, Haimen, China) and observed under an inverted fluorescence microscope (200 ×).

### In vitro biocompatibility of MB_VEGFR2_

Cell cytotoxicity: The cytotoxicity of MB_VEGFR2_ was quantitatively analyzed via the CCK-8 assay (MedChemExpress, USA). In brief, HUVECs were seeded in 96-well plates at a density of 1 × 10^4^ cells per well and incubated for 24 h before treatment. Then the cells were treated with MB_Con_ or MB_VEGFR2_ at various concentrations (10^7^/ mL, 10^6^ / mL, 10^5^/ mL and 10^4^/ mL) for another 24 h at 37 °C. Subsequently, 10 μL CCK-8 solution was added and incubated with cells for 2 h. Finally, the inhibition rate of the samples was measured via the absorbance intensity at 450 nm.

Hemolysis Assay: Analysis of in vitro hemolysis of MB_VEGFR2_ was conducted as previously described [45]. 1mL fresh blood obtained from BALB/c nude mice by heart puncture was diluted with 2 mL PBS. Red blood cells (RBCs) were separated from the serum thrice by centrifugation (2000 rpm, 10 min). After washing three times, RBCs were then diluted with 10 mL PBS. A suspension of RBCs (100 μL) was incubated with 400 μL of PBS (negative control), distilled water (positive control), MB_Con_ and MB_VEGFR2_ at different concentrations separately (10^8^/ mL, 10^7^/ mL, 10^6^/ mL, 10^5^/ mL and 10^4^/ mL) at 37 °C for 2 h. After centrifugation for 10 min, the hemolysis images were collected, and the absorbance of supernatants (100 μL) at 410 nm was measured using a microplate reader (BIOTEK, USA).

### In vivo superb microvascular imaging

After induction of anesthesia, the mice were positioned supine on a homeothermic blanket at 37 °C. Superb Microvascular Imaging (SMI) was used for the noninvasive assessment of microvessels. SMI imaging was performed using the Aplio 500 system (Canon Medical Systems Corp., Tokyo, Japan) with a 5- to14-MHz linear transducer. Vascular images were obtained from the region of interest (ROI), and the image parameters for SMI were as follows: velocity scale < 2 cm/sec; dynamic range, 21 dB; and frame rate, 27–60 frames/sec. The plane with the most extensive vasculature was selected as the representative image for evaluation. We evaluated the vascular index (VI, %) as a quantitative parameter. The VI indicates the ratio between the pixels for the Doppler signal and those for the whole lesion. An average of three repeated measurements was documented for each mouse tumor.

### In vivo molecular ultrasound imaging

USMI was performed for each mouse. USMI images of mice tumors were acquired using a 10L4 high-frequency (2.9–9.9 MHz) probe of a clinical US imaging system (Acuson Sequoia ultrasound system, Siemens, Erlangen, Germany), and imaging parameters as follows: Low frequency, a gain of 0 dB, an image depth of 4 cm, an acoustic output of 0.2%, a dynamic range of 70 dB, and MI of 0.09. The US probe was placed upon the tumor region to obtain images. Mice were injected 50 μL of MB_Con_ and then MB_VEGFR2_ dissolved in PBS (4 × 10^6^ MBs, 10^7^/ mL concentration) via tail veins at 30 min intervals to allow clearance. USMI was conducted via a destruction-replenishment method as follows: After MBs injection, CEUS signals of adherent MBs and circulating MBs were continuously captured for 30 s, then all MBs in the region were rapidly destroyed by increasing MI from 0.09 to 1.40 for 1 s through a “flash” function. Subsequently, post-destruction imaging from circulating MBs was obtained for 10 s. Finally, the quantification of targeted imaging signals of both MB_Con_ and MB_VEGFR2_ was calculated by the normalized intensity difference [NIDs (%) = (predestruction signal intensity - postdestruction signal intensity) /background signal intensity × 100%] separately.

### In vivo biocompatibility of MBs

Mice were injected with 100 μL of MB_Con_, MB_VEGFR2_ or PBS, respectively (n = 6 in each group) via the tail vein (about 2 × 10^8^/ mL, 100 times the normal dosage). One day later, blood was taken, and vital organs (heart, liver, spleen, lungs and kidneys) were harvested. Serum biomarkers, including alanine aminotransferase (ALT), aspartate aminotransferase (AST), creatinine (Cr) and blood urea nitrogen (BUN), were measured using a clinical chemistry analyzer system (Hitachi 7600). Organs were fixed and underwent H&E staining.

### Histology and immunohistochemistry assays

Mice were sacrificed, and tumors were collected for histological analysis. Samples were fixed in 4% formalin for 24 h and embedded in paraffin. Then, Hematoxylin/eosin (H&E) staining of tumor tissue sections was performed after deparaffinization.

Immunohistochemical staining was performed to determine tumor neovascularization and the CD34-microvessel density (MVD). After deparaffinization with xylene and alcohol, tumor tissues were incubated in 10 mM citrate buffer (pH 7.4) for 10 min at 90℃ for antigen retrieval. 0.3% H_2_O_2_ in methanol was added to the sections for 30 min at 4℃ to inactivate the endogenous peroxidases. The sections were incubated with 0.5% Triton for 10 min and then treated with 10% normal goat serum for 1 h at room temperature for antigen blocking. CD34 (Abcam, Cambridge, MA, diluted to 1/200) antibodies were added to the sections for 1 h at 37℃. Finally, after being washed with PBS, all sections were incubated with the horseradish-conjugated secondary anti-rabbit antibody for 1 h. The immunoreactivity on the tissue sections was visualized using the peroxidase substrate DAB. The nuclei were stained with hematoxylin. The histopathological evaluation was performed by a pathologist (with 9 years of clinical experience) blinded to the USMI results. Briefly, slides were examined carefully at a low power magnification (× 40) to identify the areas with the highest density of capillaries and small vessels. 5 representative fields (400 ×) of each sample were counted, and the average was obtained. The value of MVD was analyzed based on the criteria documented by Weidner et al. [46]. In addition, the total integrated optical density (IOD) were determined using the IPP 6.0 imaging software (Media Cybernetics, Silver Spring, MD, USA).

### Statistical analysis

Statistical analyses were conducted using SPSS v22.0 (SPSS Inc., USA). The Kolmogorov-Smirnov test was performed to assess the normality of the data. Normally distributed continuous data were expressed as mean ± standard deviation and compared by two-sided Student t-test (two groups) or ANOVA test (multiple groups). Otherwise, variables were expressed as the median and interquartile range, compared using the Kruskal-Wallis test. Linear correlation analyses were performed by GraphPad Prism8. *P* values (two-tailed) < 0.05 were statistically significant.

## Electronic supplementary material

Below is the link to the electronic supplementary material.

Supplementary Material 1

## Data Availability

Not applicable.
