# Supplementary material for: VEGFR2 targeted microbubble-based ultrasound molecular imaging improving the diagnostic sensitivity of microinvasive cervical cancer
Source: J Nanobiotechnology. 2023 Jul 12;21:220. doi: 10.1186/s12951-023-01984-2 (PMC10337210; doi:10.1186/s12951-023-01984-2)
Supplement: Supplementary file 1 — Supplementary Material 1 [file 12951_2023_1984_MOESM1_ESM.docx]

**VEGFR2 Targeted Microbubble-based Ultrasound Molecular Imaging Improving the Diagnostic Sensitivity of Microinvasive Cervical Cancer**

Junlin Zhong^1†^, Manting Su^1†^, Ye Jiang^2^, Licong Huang^1^, Ying Chen^1^, Zhuoshan Huang^3^, Xinling Zhang^1*^

^1^Department of Ultrasound, The Third Affiliated Hospital of Sun Yat-sen University, No. 600 Tianhe Road, Guangzhou, Guangdong 510630, China

^2^Department of Pathology, The Third Affiliated Hospital of Sun Yat-sen University, No. 600 Tianhe Road, Guangzhou, Guangdong 510630, China

^3^Department of Cardiovascular Medicine, The Third Affiliated Hospital of Sun Yat-sen University, No. 600 Tianhe Road, Guangzhou, Guangdong 510630, China

†Junlin Zhong and Manting Su contribute equally to the work.

*Correspondence should be addressed to: zhxinl@mail.sysu.edu.cn for Xinling Zhang.

**Keywords:** microinvasive cervical cancer, noninvasive diagnosis, molecular ultrasound imaging, VEGFR2


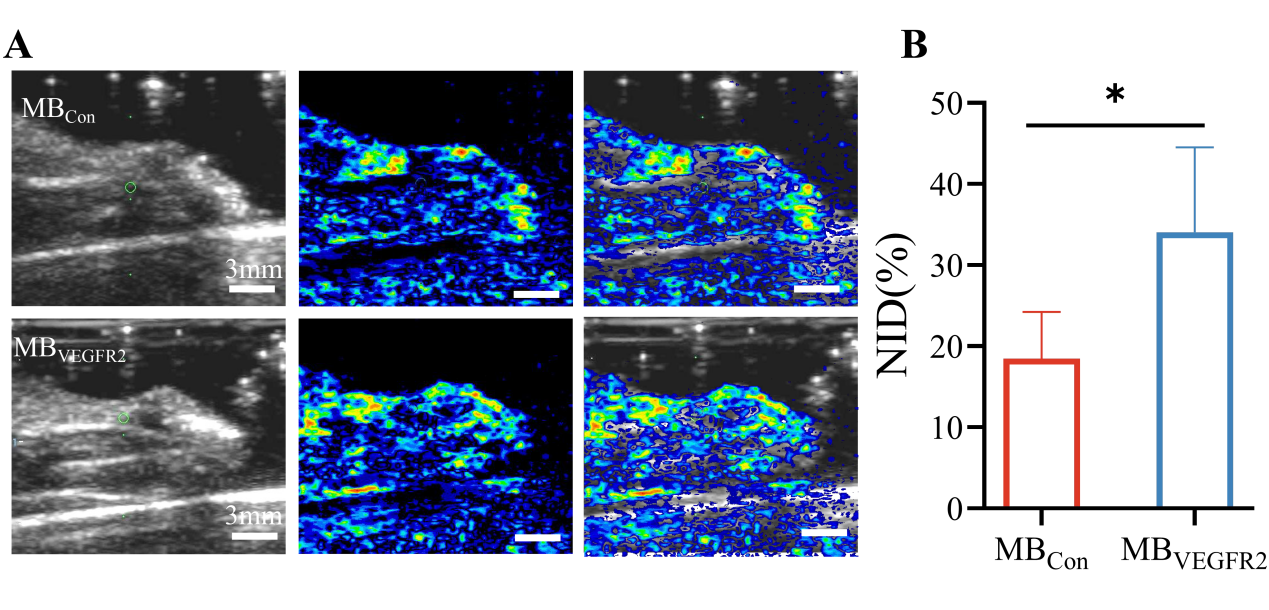


**Figure S1:** Representative ultrasound images in B-mode (gray) and molecular ultrasound images (color coded) after administrations of MB_Con_ **(A)** and MB_VEGFR2_ **(B)** in mouse cervical cancer model (n=5, * *P* < 0.05).

**
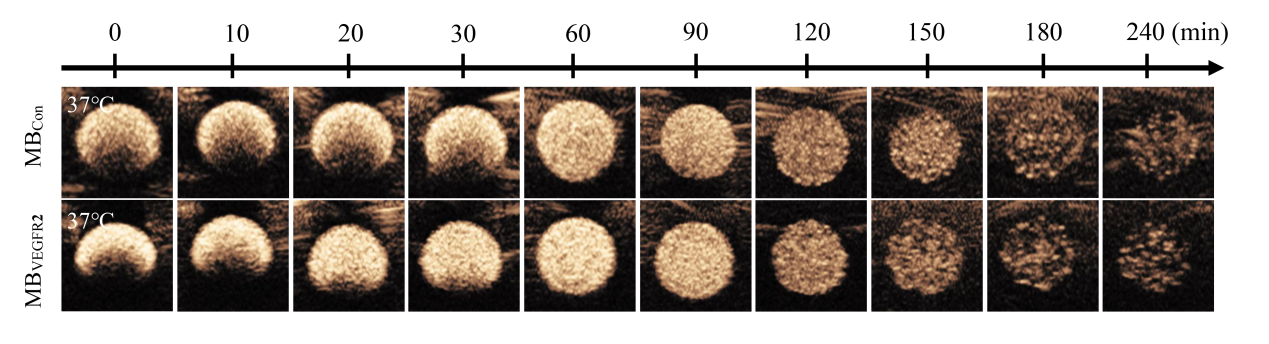
**

**Figure S2:** The stability and imaging ability of MB_Con_ and MB_VEGFR2_ at 37℃.

**
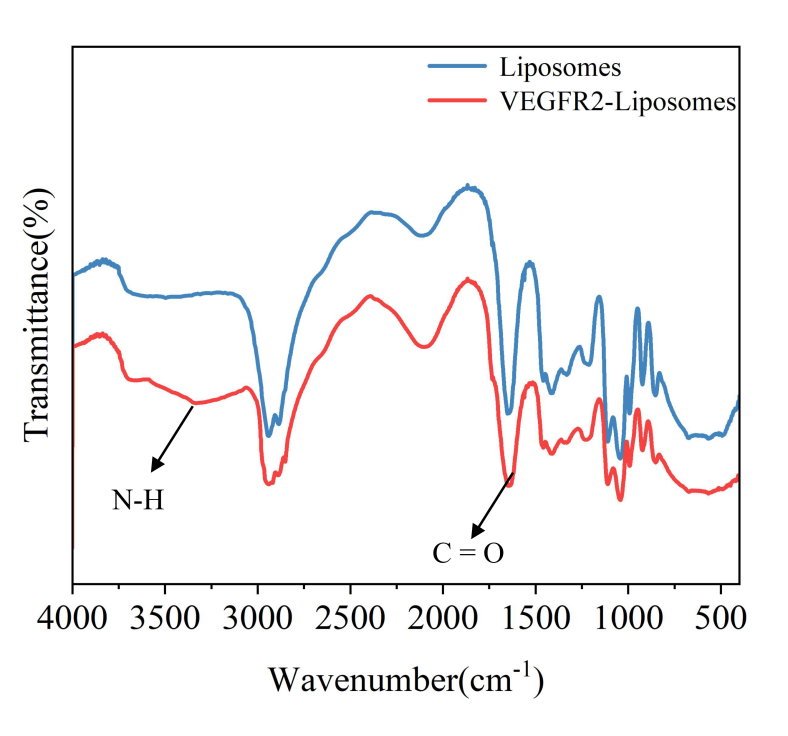
**

**Figure S3**: The Fourier-transform infrared (FTIR) spectra of liposomes and VEGFR2-liposomes
